# Low dose of rFSH [100 IU] in controlled ovarian hyperstimulation response: a pilot study

**DOI:** 10.1186/1757-2215-7-11

**Published:** 2014-01-21

**Authors:** Caio Parente Barbosa, Emerson Barchi Cordts, Andrea Couto Costa, Renato de Oliveira, Marina Acosta de Mendonça, Denise Maria Christofolini, Bianca Bianco

**Affiliations:** 1 Human Reproduction and Genetics Center, Faculdade de Medicina do ABC, Santo André, SP, Brazil

**Keywords:** Assisted reproduction treatment, Controlled ovarian hyperstimulation, Recombinant FSH, Infertility, Oocyte

## Abstract

**Background:**

The initial dose of recombinant Follicle Stimulating Hormone [rFSH] to be used in assisted reproduction treatment depends on several factors, mainly the cause of the infertility and the patient’s age. For young patients [≤35 years] usually an initial dose of around 150 IU of rFSH is recommended, but there are no studies proving that this should actually be the standard initial dose. We aimed to report the experience of a low-cost Human Reproduction Center where a dose of 100 IU of rFSH was used for controlled ovarian hyperstimulation [COH].

**Findings:**

An observational prospective study was performed on 212 women aged ≤38 years old that underwent high-complexity assisted reproduction treatments. The patients’ infertility was mainly caused by tuboperitoneal, idiopathic or male factors. Controlled ovarian stimulation was performed using 100 IU of rFSH. Regarding the COH, 53.8% of the patients presented a satisfactory response, 25.9% low response, 14.2% hyper-response, and 6.1% developed ovarian hyperstimulation syndrome. Of the 55 patients with poor response, 20 started a new cycle with an initial dose of 200 IU of rFSH; 65% showed a satisfactory response, 10% a poor response, 20% a hyper-response, and 5% developed OHSS.

**Conclusion:**

The initial dose of 100 IU of rFSH was considered adequate for controlled ovarian hyperstimulation, meeting the aim to reduce the costs of the assisted reproduction treatment.

## Background

Conjugal infertility is characterized by the absence of spontaneous pregnancy after the minimum period of twelve months, with the practice of regular and unprotected intercourse [1]. Although infertility is not a physical problem and does not threaten the life of the individual, marital infertility is related to emotional, mental and social problems, as the act of procreation is one of the goals and desires of the human [2]. Studies conducted by the British Association of Counselling in Infertility and the Royal College of Obstetricians and Gynecologists demonstrated that infertility is related to low productivity and the loss of financial reserves for that country [3]. Approximately 20% of adults of reproductive age face difficulties to conceive. In Brazil, data of the Ministry of Health considered to be underestimated reported the existence of at least 278,000 individuals affected by infertility [4], however it is believed that approximately 30,000,000 people in Brazil are infertile.

Since the birth of the first in vitro fertilization [IVF] baby almost 30 years ago, dramatic developments have occurred in assisted reproductive technology. The approach of maximizing pregnancy rates per cycle has led to very complex and costly ovarian hyperstimulation protocols. Ovarian stimulation has been applied with the aim of increasing the number of oocytes in order to compensate for inefficiencies of the IVF procedure, enabling the selection of one or more embryos for transfer [5]. In the context of improved laboratory performance, the need for a large number of oocytes as an integral part of a successful IVF program may be questioned [6].

Currently used medication protocols for ovarian stimulation are complex and expensive, and can be associated with negative effects such as ovarian hyperstimulation syndrome [7] and impaired luteal phase [6]. Thus, there has been increasing interest in identification and the relative performance of tests of ovarian reserve prior to embarking on controlled ovarian stimulation [8].

Several parameters have been postulated as predictors of ovarian response. Because ovarian function cannot be measured directly, the use of serum markers [FSH (Follicle Stimulation Hormone), inhibin B, 17-β-estradiol and anti-Müllerian hormone (AMH)] and/or ultrasound variables [ovarian volume, measurement of antral follicles, ovarian stromal blood flow] have been proven useful although limited [8]. These markers do not reflect the complex follicular dynamics and none of them shows strong correlation with the population of primordial follicles that remain in the gonad. In other words, these tests do not reveal the cohort of inactive follicles responsible for the continuation of ovulatory cycles or reproductive potential [9].

In the same context, the initial dose of rFSH that should be used depends on several factors, mainly the cause of the infertility and the patient’s age. According to Devroey et al. (1998) [10], the initial dose of FSH should be around 150 IU or 225UI for young patients [<35 years] undergoing assisted reproduction treatment of high complexity, to be adjusted according to the ultrasound control [antral follicle counting] and/or dosage of plasma estradiol, but so far there are no studies proving that this should actually be the standard initial dose.

Considering the lack of data concerning the recommended initial dose of rFSH for patients undergoing assisted reproduction treatment, we report here the experience of a low-cost Human Reproduction Center where the dose of 100 IU of rFSH has been used for controlled ovarian hyperstimulation.

## Material and methods

### Patients

An observational prospective study was performed on 212 women [mean age 31.6 ± 3.4 years old] who underwent high-complexity assisted reproduction treatments at Instituto Ideia Fértil - Centro de Reprodução Humana e Genética da Faculdade de Medicina do ABC, Santo André/SP, Brazil. The inclusion criteria were: tube peritoneal, unexplained or male factor infertility, age ≤38 years old, serum FSH [≤10.0 mIU/ml], TSH [<4mIU/L] and prolactin [<25 ng/ml] within normal limits, both ovaries present and without any morphological abnormalities, normal ovulatory cycles [25 to 35 days], body mass index [BMI] ≤30, no history of inadequate ovulatory response [poor responder] and no evidence of endocrine diseases such as hyperprolactinemia, thyroid dysfunction or polycystic ovary syndrome. Exclusion criteria were: age >38 years old, high-complexity ovulation induction protocols with rFSH starting dose >100 IU, low-complexity protocols, patients with polycystic ovary syndrome, moderate/severe endometriosis [grades III and IV], previous history of ovarian surgery and/or radiation/chemotherapy.

The investigation into the cause of infertility included a hormonal and biochemical profile, testing for sexually transmitted diseases, imaging examinations, investigation of genetic and/or immunological abnormalities, and semen analysis of the partner, hysterosalpingography, hysteroscopy and laparoscopy [performed in all women up to 36 years of age and also in patients over 36 whenever there were symptoms or abnormalities on the imaging examinations]. If no abnormalities were found in these exams, the infertility was classified as unexplained.

Anatomic tubal abnormalities preventing the proper functioning of the tubes, such as tubal obstruction, functional changes caused by pelvic inflammatory disease, endometriosis, or previous tubal surgery were considered tubeperitoneal factors. These abnormalities were diagnosed by hysterosalpingography and/or laparoscopy.

According to the criteria of the World Health Organization [WHO, 2010] [11], when a patient’s partner presented an initial concentration of less than 15 million sperm/ml, 5 million/mL rapid progressive after sperm processing, or asthenospermia [less than 40% of motile spermatozoa considering rapid progressive or nonprogressive or less than 32% if we consider only the rapid progressive sperms], the case was classified as male factor infertility.

Clinical and laboratory data were collected only after explaining the objectives of the study and obtaining a signed informed consent form, as approved by the local Research Ethics Committee.

### Ovarian stimulation protocol

To the patients who met the inclusion criteria an initial daily dose of 100 IU of rFSH was administered for 10 days, starting on the second day of menstruation. As of the 6th day and until the 10th day, the antagonist was also administered. Between day 10 and 11, when the follicles reached a diameter of approximately 17 mm, as determined by transvaginal ultrasound, the patients were given human chorionic gonadotropin [hCG], and on day 13 oocyte retrieval was performed.

Patients who did not present a good response within the first eight days, i.e., whose follicles did not show satisfactory growth [at least 14 mm or did not grow at least 4 follicles over 10 mm] for further treatment, had their treatment cycles canceled. A new cycle can be started with the same or a higher dosage, according to medical indication.

With regard to the controlled ovarian stimulation we considered: i] Ovarian hyperstimulation syndrome [OHSS], characterized by multiple ovarian follicles [≥20 follicles] accompanied by ≥4000 IU of serum estradiol and possible clinical symptoms such as ascites, hematological changes [hemoconcentration], pleural effusion, and liver and/or coagulation abnormalities according to the classification proposed by Golan et al. [1989] [12]; ii] Hyperresponse, when after 6 days of ovarian stimulation with gonadotropins there was the development of ≥12 ≤ 19 follicles, without clinical symptoms of OHSS; iii] Poor response, when after 6 days of ovarian stimulation with gonadotropins only up to 3 follicles smaller than 14 mm developed; and iv] Satisfactory response, when after 6 days of ovarian stimulation with gonadotropins 4 to 12 follicles larger than 14 mm had developed.

## Results

The study included 212 patients who underwent high-complexity assisted reproduction treatments, of which 53.8% [114/212] presented a satisfactory response to controlled ovarian hyperstimulation, 25.9% [55/212] were classified as poor responders, 14.2% [30/212] showed a hyper-response, and 6.1% [13/212] developed ovarian hyperstimulation syndrome [OHSS], as shown in Table 1.

**Table 1:** Classification of patients according to their response to controlled ovarian hyperstimulation with an initial dose of 100 IU of rFSH

**Response to COH**	**Frequency[n]**	**Percentage [%]**	**MI follicles [Mean ± SD]**	**MII follicles [Mean ± SD]**
Satisfactory	114	53.8%	0.184 [0.487]	5.561 [1.933]
Poor	55	25.9%	0.036 [0.187]	1.727 [1.119]
Hyper	30	14.2%	0.966 [1.516]	10.5 [2.642]
OHSS	13	6.1%	0.307 [0.605]	16.0 [5.857]

COH - controlled ovarian hyperstimulation, OHSS – Ovarian hyperstimulation syndrome.

Considering the patients with poor response, 20 out of 55 started a new treatment cycle with an initial dose of 200 IU of rFSH, and 65% [13/20] showed a satisfactory response, 10% [2/20] a poor response, 20% [4/20] a hyperresponse, and 5% [1/20] developed OHSS, as shown in Table 2.

**Table 2:** Classification of patients with poor response to the initial dose of 100 IU of rFSH according to their response to controlled ovarian hyperstimulation with an initial dose of 200 IU of rFSH in a new treatment cycle

**Response to COH**	**Frequency [n]**	**Percentage [%]**	**MI follicles [Mean ± SD]**	**MII follicles [Mean ± SD]**
Satisfactory	13	65%	0.538 [0.929]	5.46 [1.677]
Poor	2	10%	0	1.5 [1.576]
Hyper	4	20%	1.25 [2.165]	8.5 [2.179]
OHSS	1	5%	3.0	14.0

COH - controlled ovarian hyperstimulation, OHSS – Ovarian hyperstimulation syndrome.

## Discussion

*In vitro* fertilization is a complex multistep process that comprises collection of oocyte-containing follicles after controlled ovarian hyperstimulation with FSH, oocyte fertilization, embryo development, embryo transfer to the uterus, and implantation. All these steps are critical for successful IVF. However, the initial critical step of this complex procedure is the COH whose aim it is to safely obtain a high number of mature oocytes, so as to allow the selection of the most viable embryo for transfer. Both quantitative and qualitative factors in oocyte production have a high influence on the IVF outcome. The significant inter-individual variability regarding COH with rFSH is one of the most challenging issues in IVF treatment. The goal is to transfer a single embryo and thus reduce the risk of multiple pregnancies, the main complication of IVF [13,14].

Since both practitioners and patients believed that the replacement of several embryos increased the success rate, multiple embryo replacement was the rule. Very slowly one realized that this practice had considerable side effects. The price to be paid for the multiple births is enormous, both in financial and health terms, but also in terms of psychological suffering [15,16]. An important move away from this model occurred only a few years ago with the introduction of single embryo transfer [17,18].

The process that started with single embryo transfer should eventually result in a global ’patient-friendly’ approach. Patient-friendly assisted reproductive treatment includes four different aspects or criteria: cost-effectiveness, equity of access, risk minimization and burden minimization for the patients [19].

In 1996, Edwards et al. were the first to express concern with regard to contemporary ovarian stimulation approaches for IVF and called for the use of a milder stimulation protocol [20]. The aim of mild stimulation is to develop safer and more patient-friendly protocols in which the risks of treatment are minimized [7,19,21-23].

The term “low-cost IVF” was coined by Professor Alan Trounson at a World Health Organization meeting in Geneva in 2001. The concern of the World Health Organization had always been to promote advances in health care in low resource economies. A milder stimulation protocol offers the same advantages as natural-cycle IVF, but has higher efficacy by recruiting few dominant follicle in addition to a many codominant follicles that do not develop spontaneously in a natural cycle [7,24]. However, there are reservations about the efficacy of a low-dose approach, including lower pregnancy rates compared with conventional IVF, in addition to a lower profit for IVF centers [6].

In underdeveloped or developing countries, infertility treatment can involve more complex socioeconomic issues, raising two main points: the problem of superpopulation, about which it is argued that this should be the focus of family planning programs; and, in view of the high cost of an assisted reproduction treatment compared to the low income of the population, that this income should be directed towards higher priorities. However, even in these countries, it is observed that the consequences of infertility without a possibility of treatment vary from a period of anguish to social isolation, murder and even suicide [25].

As older health care demands are controlled, new needs arise in our days, bringing about increasing costs and the issue of universal access. In between the public and the private sphere, the third sector arises in the process of remodeling the institutional managing ways as a combination of the several kinds of arrangements between the State and the civil society, so as to implement and co-manage public policies and to face the different manifestations of the Brazilian social issues. Distant from a philanthropic and charitable perspective, this “group” is organized with the purpose of warranting the respect of the rights resulting from the Constitution of 1988 and the Organic Laws they entail.

Infertility treatment arises as an emerging need, as it is estimated that in Brazil over 278,000 couples face difficulties in conceiving a child at some point of their reproductive age. The development of new assisted reproduction technologies and the search for success in the treatment of human infertility have generated an increasing demand for such treatments in the public sector, the third sector and the private services, even at a significantly high cost.

Countless questionings have been brought up ever since these techniques have been incorporated to the arsenal of medical procedures made available to the population, and their use is more and more common in social organizations that aim to ensure, by means of an integrated set of actions, the constitutional rights, among which health as a qualification of the civil right to life.

The technological advances bring about new questionings, such as the role of the State in the access of the less favored population to modern treatments and all the other necessities of a Third-World country. A health care system that offers equitable access to basic health care services is only viable when both patients and physicians realize that the interests of the individual patient and of the social system must be balanced. This implies that patients do not have a right to the most effective treatment [regardless of the cost] but only to the most cost effective treatment [19].

Based on the cost of the medication used for controlled ovarian stimulation, manufactured by several pharmaceutical industries, the use of an initial dose of 100 IU of rFSH represents a saving of approximately 45% of the total cost of the treatment. This saving may make it viable to offer low-income populations a possibility to have children and make their dream of a family come true.

## Conclusion

In conclusion, the initial dose of 100 IU of rFSH was considered adequate for controlled ovarian hyperstimulation, aiming to reduce the costs of the assisted reproduction treatment, considering 'patient-friendly’ assisted reproductive treatment.

## Competing interests

The authors declare that they have no competing interests.

## Authors’ contributions

CPB; EBC and BB conceived study design. EBC; ACC and Rde Oliveira performed the data collection and analysed data. CPB, MA de Mendonça, DMC and BB interpretation the data. All authors were involved in literature search, writing the paper and had final approval of the submitted and published versions. All authors read and approved the final manuscript.
